# Vitamin E deficiency in South Asian population and the therapeutic use of alpha-tocopherol (Vitamin E) for correction of anemia

**DOI:** 10.12669/pjms.346.15880

**Published:** 2018

**Authors:** Tanveer Jilani, Mohammad Perwaiz Iqbal

**Affiliations:** 1 *Dr. Tanveer Jilani, PhD. Department of Biological and Biomedical Sciences, Aga Khan University, Karachi, Pakistan*; 2 *Prof. Dr. Mohammad Perwaiz Iqbal, PhD. Department of Biological and Biomedical Sciences, Aga Khan University, Karachi, Pakistan*

**Keywords:** Alpha tocopherol, Vitamin E deficiency, South Asian population, Anemia

## Abstract

Mild to moderate vitamin E deficiency because of inadequate consumption of vitamin E-rich foods and intestinal fat malabsorption is common in growing children, women of reproductive age and elderly South Asian population. Severe vitamin E deficiency may lead to peripheral and motor neurodegenerative diseases (e.g ataxia and motor skeletal myopathy), impaired immune response and free radical-induced hemolytic anemias. Vitamin E insufficiency and/or deficiency status in the general Pakistani population has not been sufficiently investigated. Moreover, there are challenges in determining vitamin E status in apparently healthy humans due to variations in their age, sources of consumed vitamin E and plasma lipid levels. Oxidative stress-induced reactive oxygen species have been shown to cause ineffective erythropoiesis and enhanced lysis of erythrocytes in some of the experimental animals and humans. Several studies on patients with various types of inherited hemolytic anemias, chronic renal disease, premature low birth infants and apparently healthy humans have shown that vitamin E might be therapeutically effective in the prevention and/ or treatment of anemia in these subjects.

## INTRODUCTION

### Vitamin E Deficiency in South Asia

Vitamin E is lipophilic vitamin which is widely distributed in the commonly consumed food items such as oils, vegetables, seeds, nuts and fruits, and therefore its deficiency in general population is considered to be uncommon, especially among the technologically advanced countries.^1^ However, in the developing countries of South Asia, mild vitamin E deficiency is reported to be present in the undernourished populations with associated morbidities.^1^,^2^ Studies carried out in the West have shown that the normal range of plasma levels of vitamin E (α-tocopherol) in adult population is 5 µg/ml (11µmol/l) – 15 µg/ml (33 µmol/l).^2^

Human vitamin E deficiency (plasma α-tocopherol level of < 5 μg/ml) has been found to be often associated with chronic malabsorption of fat, mutations in the α-tocopherol transfer protein and with birth of premature low-birth weight infants.^3^ Severe or chronic vitamin E deficiency may lead to peripheral neurodegenerative diseases, skeletal myopathies and increased vulnerability to infections because of compromised immune function, especially among the older people.^4^ A couple of reports from South Asia have shown association of severe deficiency of vitamin E with ataxia.^5^,^6^ Moreover, it had been suggested in some of the earlier human studies that vitamin E deficiency could also be associated with anemia due to reduced erythrocyte life span as a result of increased vulnerability to free-radical-induced erythrocyte membrane lysis.^7^ The relationship of vitamin E deficiency with hemolytic anemia in premature infants has been recently highlighted again.^8^

Extensive literature search has revealed that hardly any attempt has been reported to assess serum vitamin E status in apparently healthy South Asian population. 9 In general, vitamin E deficiency is rarely seen in adults but is often found in South Asian children with certain conditions such as severe protein energy malnutrition, fat malabsorption syndromes such as cholestatic liver disease or cystic fibrosis and infections such as malaria and acute respiratory tract infection.^3^,^10^,^11^ This was supported by a study on five months to 12 years old Chinese children showing that those having vitamin E deficiency were more likely to acquire acute respiratory tract infections.^10^ Moreover, prevalence of vitamin E deficiency was reported to be 92% in 3-8 year-old Indian children suffering from protein energy malnutrition as compared to 12% in age-matched apparently healthy controls.^12^

Dietary vitamin E deficiency is common in healthy pregnant South Asian women and had also been suggested to be associated with increased chances of abortion in these women.^13^-^15^ For example, in a study in Bangladesh, 43.5% of the pregnant Bangladeshi women were found to be α-tocopherol deficient.^13^ Similarly, 98% of Indian pregnant mothers from low-socioeconomic background were found to be vitamin E deficient on the basis of low serum levels of α-tocopherol.^15^ A few studies indicated that vitamin E deficiency might be quite common in the healthy middle-aged and elderly people in the developing and underdeveloped South Asian countries because of lower intake of vitamin E-rich food items which might lead to oxidative stress-induced degenerative diseases in these people.^16^

Apart from a few case reports, no large-scale study representing Pakistani population on the status of vitamin E or plasma level of α-tocopherol has been published.^6^,^9^ Thus, finding out normal plasma/serum levels of α-tocopherol or extent of deficiency of vitamin E in Pakistani population has remained an unexplored research question. Based on few studies, it can be said that mild deficiency of vitamin E could be commonly present in Pakistani population. Poor nutrition and cooking practices in this country could be contributing to deficiency of this vitamin. Casal et al have demonstrated that foods with high vitamin E content when subjected to high temperatures would lose a significant amount of the vitamin.^17^ Since Pakistani people, in general, have the habit of overcooking vegetables, fish and other food items, the risk of decreasing vitamin E content is very significant. Thus, vitamin E insufficiency could be quite common in Pakistani population. A small study (n=81) carried out in Karachi found vitamin E deficiency (α-tocopherol levels less than 5 µg/ml or 11µmol/l) to be 4.9% in apparently healthy adults with age range 20 – 65 years.^18^ In an earlier study, dietary vitamin E deficiency had been shown to alter the erythrocyte membrane fatty acid composition in malnourished Pakistani children.^19^

### Challenges in determining vitamin E deficiency

Studies have shown that plasma vitamin E levels may vary in normal healthy individuals depending on the type of source of vitamin E intake. Plasma α-tocopherol levels were reported to be on an average 20 μmol/l in those healthy adult humans who consumed vegetable seeds, nuts and whole grains. On the other hand, levels greater than 30 μmol/l have been usually observed in healthy individuals whose main sources of vitamin E were fortified foods or vitamin E supplements.^20^ Moreover, it has been proposed that with normal increasing age, the α-tocopherol level may also increase due to increase in its plasma carrier proteins as a result of increase in plasma lipid concentrations which may thus affect the appropriate assessment of vitamin sufficiency and/ or deficiency status.^20^ Therefore, for accurate determination of vitamin E status, age, plasma lipid levels, consumption of vitamin E rich food items and adipose tissue (which functions as a store of vitamin E and releases it in increased metabolic demand) must be taken into account. 11

### Vitamin E and anemia

### Effect of oxidative stress on erythropoiesis

When the normal balance between the available quantity and functioning of pro-oxidants and antioxidants gets altered, it can lead to enhanced cellular oxidative stress. Oxidative stress is known to produce Reactive Oxygen Species (ROS) which are highly reactive free radicals which could rapidly interact with cellular macromolecules such as membrane lipids, proteins, polysaccharides and nucleic acids and damage the cell. This oxidative damage to erythroid cells often results in impaired red blood cell biosynthesis and may lead to premature hemolysis due to increased fragility of red cell membrane.^22^ Thus, increased oxidative stress in an individual can lead to development of anemia.

### Vitamin E as a therapeutic agent in the treatment of anemia

Some of the clinical studies on humans during the last few years have shown that antioxidants such as vitamin E could be useful in the treatment of anemia.^23^,^24^ Vitamin E is a highly potent lipophilic vitamin which can protect against the ROS by breaking the free radical-initiated chain of lipid peroxidation reactions in cell membranes and organelles. Vitamin E is the primary antioxidant in humans which maintains red blood cells intergrity.^24^ In chronic hemolytic anemia where there is excessive oxidative damage to the red blood cells, significantly decreased levels of vitamin E have been observed.^25^ In such conditions, polyunsaturated fatty acids in the red blood cell membrane undergo lipid peroxidation resulting into increased membrane fragility and lysis due to oxidative stress. Vitamin E has been shown to ameliorate hemolysis of red blood cells.^24^ A study by George and Adoke suggested that gasoline induced hemolysis of red blood cells in albino rats could be prevented by feeding them vitamin E rich diet showing the antioxidant property of this vitamin against hemotoxicity.^26^ Role of vitamin E as an erythrocyte forming factor was demonstrated in a study by Gogu et al in a murine model.^27^ Addition of α-tocopherol to mouse bone marrow cells in culture led to a dose-dependent increase in erythroid colony forming units (CFU-E). When the experiment was carried out in mice, it not only increased the number of CFU-E in bone marrow, but also improved the hemoglobin levels thereby correcting drug-induced anemia.^27^

### Effects of vitamin E treatment in patients with hemolytic anemia

The erythrocytes of patients suffering from hereditary hemolytic anemias are predisposed to increased risk of chronic oxidative stress-induced membrane lipid peroxidation of developing erythroblasts and circulating erythrocytes leading to shortened red blood cell survival. Deficiency of vitamin E is a common feature in some of these hereditary hemolytic anemias.^28^ Results of a number of studies revealed that supplementation with α-tocopherol in patients suffering from sickle cell anemia, beta-thalassemia and glucose-6-phosphate dehydrogenase deficiency significantly reduced red blood cell deformity, decreased erythrocyte osmotic fragility and premature hemolysis and thereby increased blood hemoglobin levels as compared to healthy controls.^29^-^31^

### Clinical trials of vitamin E in chronic renal failure patients

Studies conducted during the last three decades have indicated an increase in production of free radicals and/ or decrease in the functional activity of antioxidant enzymes and vitamins in patients with chronic (end-stage) renal failure.^32^, ^33^ Treatment with α-tocopherol and /or vitamin E-coated dialyzers have been shown to significantly decrease oxidative stress, inhibit red blood cell osmotic fragility and enhanced post-supplemental hemoglobin concentration in these end-stage renal failure patients.^34^, ^35^

### Clinical trials of vitamin E in premature low birth weight infants

Some of the studies conducted on premature low birth weight infants have shown abnormally high ROS-induced erythrocyte hemolysis in them. Treatment of these low birth weight infants with low to moderate doses of vitamin E was found to inhibit development of hemolytic anemia in these infants.^36^ In another study, treatment with alpha tocopherol (25 IU/day) along with iron supplementation (5 mg/kg/day) significantly improved anemia in preterm neonates.^37^

### Relationship of plasma levels of vitamin E with blood hemoglobin levels in apparently healthy South Asian adults

A study conducted by Shamim et al on apparently healthy Bangladeshi pregnant women (n=285) showed alpha-tocopherol deficiency and a positive relationship of plasma levels of α-tocopherol with hemoglobin concentration was found in this cohort.^14^ A previous study from Pakistan indicated a positive effect of α-tocopherol treatment in enhancing blood hemoglobin concentration in mildly anemic Pakistani adults.^38^ Subsequently, in a randomized controlled trial conducted by the same group in the general population of Karachi, Pakistan, a 3-month supplementation with vitamin E to mildly anemic and apparently healthy human adults was found to be effective in increasing their hemoglobin levels.^39^ In this clinical trial, 357 apparently healthy adults visiting the General Practioners’ clinics in the urban areas of Karachi were screened for mild anemia. One hundred and twenty four subjects were identified with mild anemia. They were recruited in the clinical trial with informed consent. They were randomly divided into two groups- a treatment group (n=82) and a control group (n=42). The treatment group subjects were given 400 mg vitamin E daily for a period of three months, while the subjects in the control group received placebo for the same period. After three months, their levels of hemoglobin and serum / plasma levels of ferritin, soluble transferrin receptor, folate, vitamin B_12_, erythropoietin, total antioxidant status and vitamin E were measured at baseline and endline. Results were analyzed by multiple linear regression and hemoglobin levels were found out to be significantly associated with serum levels of vitamin E.^39^ The total antioxidant status of these subjects was not significantly different between the control arm and the treatment arm of the study indicating that antioxidant property of vitamin E had little role in enhancing the hemoglobin levels in these subjects. One of the possible mechanisms of enhancing hemoglobin concentration in apparently healthy mildly anemic adults by vitamin E appears to be due to suppression of inhibition of apoptosis of human erythroid progenitor cells. This phenomenon needs to be further investigated.

### Recommendations

Vitamin E deficiency and insufficiency are not uncommon in the developing world and it is mostly seen in malnourished children. Therefore, consumption of vitamin E rich food such as, nuts (almonds, walnuts, peanuts, pistachios), seeds, fruits (mangoes, papayas, lychees), vegetables (spinach, turnip, asparagus, lettuce), fish, eggs and oils (wheat germ oil, canola oil, olive oil) is necessary to avoid morbidities and ailments associated with its deficiency.^40^ A daily intake of 12 – 15 mg of vitamin E per day is considered sufficient in normal healthy adults to provide adequate status of this vitamin i.e. serum levels of α-tocopherol around 13 µg/ml (30 µmol/l).^41^,^42^ These levels have been shown to reduce the risk of chronic diseases.^42^

## CONCLUSION

Recent reports have shown that a moderate to high vitamin E deficiency is reported in the developing countries of South Asia and it may be the cause of various neurodegenerative diseases, impaired immune response and free radical-induced hemolytic anemias. Vitamin E supplementation has the potential to be therapeutically used for preventing/and or treating some types of the human anemia due to its suggested role in enhancing erythropoiesis, decreasing the oxidative stress-induced erythrocyte lysis and thereby improving blood hemoglobin levels possibly by inhibition of apoptosis of human erythroid progenitor cells.
